# Deep Immune and RNA Profiling Revealed Distinct Circulating CD163+ Monocytes in Diabetes-Related Complications

**DOI:** 10.3390/ijms251810094

**Published:** 2024-09-19

**Authors:** Elisha Siwan, Jencia Wong, Belinda A. Brooks, Diana Shinko, Callum J. Baker, Nandan Deshpande, Susan V. McLennan, Stephen M. Twigg, Danqing Min

**Affiliations:** 1 Greg Brown Diabetes and Endocrine Research Laboratory, Sydney Medical School (Central), Faculty of Medicine and Health, Charles Perkin Centre, The University of Sydney, Sydney, NSW 2006, Australia; elisha.siwan@sydney.edu.au (E.S.); jencia.wong@health.nsw.gov.au (J.W.); callum.baker@sydney.edu.au (C.J.B.); sue.mclennan@sydney.edu.au (S.V.M.); stephen.twigg@sydney.edu.au (S.M.T.); 2 Department of Endocrinology, Royal Prince Alfred Hospital, Sydney, NSW 2050, Australia; belinda.brooks@sydney.edu.au; 3 Sydney Cytometry, The University of Sydney, Sydney, NSW 2006, Australia; d.shinko@ucl.ac.uk; 4 Sydney Informatics Hub, The University of Sydney, Sydney, NSW 2006, Australia; nandan.deshpande@sydney.edu.au; 5 NSW Health Pathology, Sydney, NSW 2050, Australia

**Keywords:** diabetes mellitus, complications, inflammation, monocytes, scavenger receptor, CD163

## Abstract

CD163, a scavenger receptor with anti-inflammatory function expressed exclusively on monocytes/macrophages, is dysregulated in cases of diabetes complications. This study aimed to characterize circulating CD163+ monocytes in the presence (D^+Comps^) or absence (D^−Comps^) of diabetes-related complications. RNA-sequencing and mass cytometry were conducted on CD163+ monocytes in adults with long-duration diabetes and D^+Comps^ or D^−Comps^. Out of 10,868 differentially expressed genes identified between D^+Comps^ and D^−Comps^, 885 were up-regulated and 190 were down-regulated with a ≥ 1.5-fold change. In D^+Comps^, ‘regulation of centrosome cycle’ genes were enriched 6.7-fold compared to the reference genome. *MIR27A, MIR3648-1,* and *MIR23A*, the most up-regulated and *CD200R1*, the most down-regulated gene, were detected in D^+Comps^ from the list of 75 ‘genes of interest’. CD163+ monocytes in D^+Comps^ had a low proportion of recruitment markers CCR5, CD11b, CD11c, CD31, and immune regulation markers CD39 and CD86. A gene–protein network identified down-regulated *TLR4* and CD11b as ‘hub-nodes’. In conclusion, this study reports novel insights into CD163+ monocyte dysregulation in diabetes-related complications. Enriched centrosome cycle genes and up-regulated *miRNAs* linked to apoptosis, coupled with down-regulated monocyte activation, recruitment, and immune regulation, suggest functionally distinct CD163+ monocytes in cases of diabetes complications. Further investigation is needed to confirm their role in diabetes-related tissue damage.

## 1. Introduction

Global diabetes incidence is rising dramatically, from approximately 536.6 million in 2021 to an estimated 783.2 million by 2045 [1]. The International Diabetes Federation has named diabetes as the largest global epidemic of the 21st century [2]. The increasing prevalence of diabetes foreshadows the rise of its associated complications, which worsen over time, leading to higher rates of mortality, morbidity, and healthcare costs [3]. Chronic low-grade systemic inflammation is a feature of diabetes and a contributing factor to the development of diabetes-related complications. Persistent inflammation induces an influx of inflammatory cells, specifically monocytes, into the tissue which contributes to the pathophysiology of complications in diabetes [4].

Recently, we reported that diabetes complication status is co-segregated with the monocyte phenotype. The number of circulating CD163+ monocytes was reported to be significantly decreased in individuals with complications compared to those without complications [5]. CD163, the hemoglobin (Hb)–haptoglobin (Hp) scavenger receptor, clears free circulating toxic Hb via Hb–Hp complexes, thereby reducing the local oxidative stress caused by reactive oxygen species. The CD163+ monocyte acts as a scavenger with anti-inflammatory functions, resolving systemic or local inflammation and ultimately protecting the tissues from damage [6,7]. In the same study, it was observed that alterations of the CD163+ monocytes were associated with circulating levels of matrix metalloproteinases (MMPs) and cytokines, indicating a potential regulatory interaction between these proteins and CD163+ monocytes [5].

Additionally, the tissue presence of CD163+ macrophages in various diabetes complications was examined in a recent systematic review and was found to differ between individuals with and without diabetes complications [8]. In individuals with diabetes and cardiovascular disease, CD163+ cells were decreased in the atherosclerotic plaques compared to individuals with cardiovascular disease without diabetes [9]. In renal complications, CD163+ cells in the glomeruli were reported to be positively correlated with the progression of nephropathy in individuals with diabetes [10].

While CD163+ monocytes/macrophages have been studied across various diabetes complications, how CD163+ monocytes are dysregulated in diabetes complications is unclear. Therefore, we aimed to comprehensively characterize circulating CD163+ monocytes, focusing on their functional profile and regulatory mechanisms, in individuals with long-duration diabetes, with or without complications. We hypothesize that there exists a distinct profile of CD163+ monocytes between these two groups, particularly emphasizing the presence of dysfunctional circulating CD163+ monocytes in individuals with complications.

## 2. Results

### 2.1. Clinical Characteristics of Individuals with Diabetes with or without Complications

The clinical characteristics of D^+Comps^ and D^−Comps^ are presented in Table 1. The two study groups were well matched with no significant difference in age, duration of diabetes, BMI, HbA_1c_, or lipid profile. Individuals with D^+Comps^ showed a trend towards worse kidney function, although this difference was not significant. They also had lower diastolic blood pressure, and more of them were on insulin therapy (both *p* < 0.05). All individuals in D^+Comps^ exhibited retinopathy. Among them, three had only microvascular complications, while another three had both microvascular and macrovascular complications, as detailed in Supplementary Table S1.

### 2.2. RNA Profiling of CD163+ Monocytes

**Figure 1:**
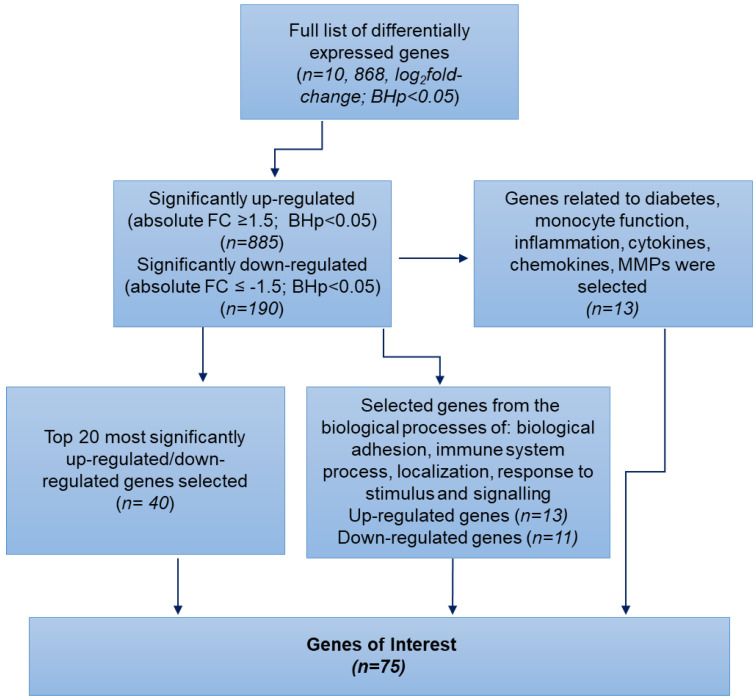
A flow-chart outlining the approach for identifying significant and relevant differentially expressed genes between individuals with long-term diabetes and complications (D^+Comps^) and those without complications (D^−Comps^). The top 20 most significantly up- and down-regulated genes were combined with selected genes identified from functional characterization on PANTHER, as well as manual searching for genes related to monocyte function, cytokines, chemokines, or MMPs, to generate a final list of ‘genes of interest’.

The Limma-Voom analysis identified a total of 10, 868 differentially expressed genes (DEGs) between D^+Comps^ and D^−Comps^ based on log_2_ fold change, with the significance adjusted using the Benjamini–Hochberg method (BHp < 0.05), as shown in Figure 1. Further analysis of these DEGs, based on an absolute fold change (FC) ≥ 1.5, revealed 885 significantly up-regulated genes (FC ≥ 1.5, BHp < 0.05), and 190 significantly down-regulated genes (FC ≤ −1.5, BHp < 0.05). This included mRNA, microRNA (miRNA), tRNA, pseudogenes, antisense RNA, and long non-coding RNAs (IncRNAs).

#### 2.2.1. Gene Over-Representation Analysis

The PANTHER over-representation test found that the genes associated with the ‘regulation of centrosome cycle’ were enriched 6.7-fold in D^+Comps^ when compared to the reference genome (false discovery rate FDR < 0.05); these included *PLK2, CENATAC, RBM14, CCNL2, KAT2A, CCNL1, XRCC3, CEP295NL, CEP131, CDK1B,* and *CDK11A*. 

#### 2.2.2. The List of ‘Genes of Interest’

**Figure 2:**
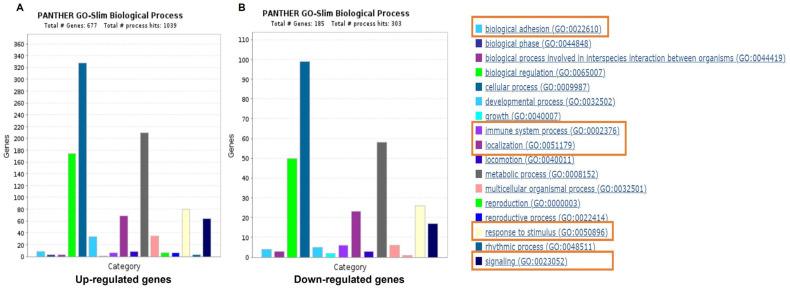
Functional characterization of up- and down-regulated genes (FC > 1.5, BHp < 0.05) in D^+Comps^ compared with D^−Comps^ according to biological processes. The orange box indicates processes that were selectively screened for genes related to our processes of interest.

As per Figure 1, the top 20 most up-regulated and down-regulated DEGs were compiled into the list of ‘genes of interest’ (GOIs) from the genes with an FC ≥ 1.5. Additionally, the list of up- and down-regulated genes was input into PANTHER to identify those associated with biological processes such as biological adhesion, immune system process, localization, and responses to stimulus and signaling (Figure 2). This screening revealed a total of 13 up-regulated GOIs related to diabetes, monocyte function, inflammation, cytokines/chemokines, and MMPs, including *ZAP70, CD5, NLGN3, MAPK81P3, MAPK13, IL11RA, ZBTB17, TRAF1, TRPV1, ZBTB25, NFKB2, IRS2* and *TRAF31P2-AS1* and 11 down-regulated genes, including *EMB, CD86, TLR4, NCK1, CD244, CD1C, PALLD, DSC2, TGFA, CX3CR1,* and *PPBP* (Figure 1).

**Figure 3:**
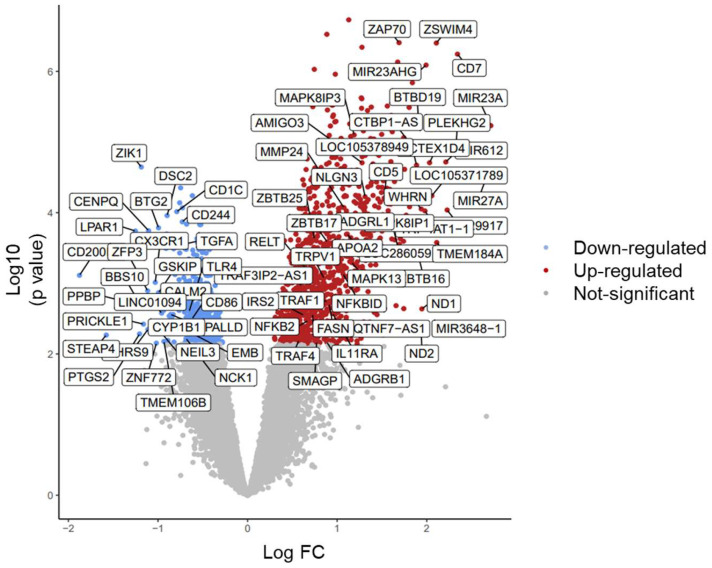
A volcano plot illustrating the selected 75 ‘genes of interest (absolute FC ≥ 1.5 and FC ≤ −1.5, BHp < 0.05).

In addition, the DEGs were rescreened based upon their functions related to diabetes, monocyte function, inflammation, cytokines/chemokines, and MMPs. This analysis revealed a further 13 up-regulated genes (FC ≥ 1.5, BHp < 0.05), which included *MAPK81P1, LOC286059, AMIGO3, APOA2, ADGRL1, MMP24, NFKBID, CIQTNF-AS1, ADGRB1, SMAGP, FASN, RELT*, and *TRAF4*. There were no further down-regulated genes found. This screening process resulted in the final list of 75 GOIs listed in Supplementary Table S2 and in a volcano plot (Figure 3). A total of 46 GOIs were up-regulated, of which the microRNAs *MIR27A, MIR3648-1,* and *MIR23A* had the greatest increase in D^+Comps^ (FC > 6-fold, BHp < 0.01, BHp < 0.05, BHp < 0.01, respectively). There were 29 down-regulated GOIs, of which *CD200R1* was the most reduced (FC −3.7-fold, BHp < 0.05). 

#### 2.2.3. Gene Interaction Network Analysis

**Figure 4:**
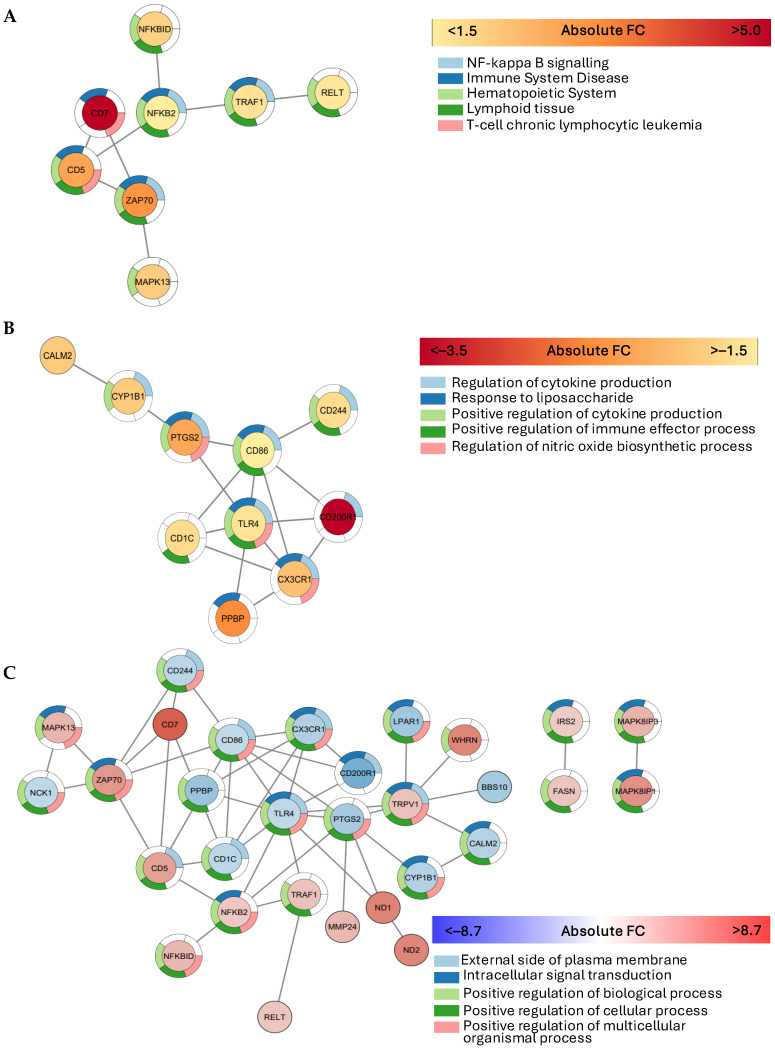
Gene interactions network illustrating the largest connected network of genes in (**A**) up-regulated genes, (**B**) down-regulated genes, and (**C**) combined up- and down-regulated genes in individuals with long-term diabetes and complications (D^+Comps^) compared to those without complications (D^−Comps^). Genes were considered significantly up- or down-regulated if the fold change was ≥1.5, and significance determined at BHp < 0.05. The color of the circular node indicates the intensity of the fold change, confirmed by the legend below. The sectioned multicolor outline of each node indicates the involvement of respective genes with the top five most enriched GO categories including pathways in the Kyoto Encyclopaedia of Genes and Genomes (KEGG).

To further understand the potential functional associations between the genes in the curated list of GOIs, interactional PPI networks were created for up-regulated GOIs (A), down-regulated GOIs (B), and all GOIs (C), as shown in Figure 4. Within the 46 up-regulated genes from the CD163+ monocytes in D^+Comps^, the search tool for the retrieval of interacting genes (STRING) identified a network of 8 nodes and 8 edges including *RELT, TRAF1, NFKB2, NFKBID, CD5, CD7, ZAP70,* and *MAPK13,* which contained interconnected genes relating to T cell function such as *RELT, NFKBID, CD5, CD7,* and *ZAP70* (Figure 4A). *CD5* was the ‘hub’ gene, with 3 edges connected to 3 different nodes, and *CD7* was the most up-regulated gene by 5.1-fold. Within the 29 down-regulated genes, STRING identified a network of 10 genes and edges including *CALM2, CYP1B1, PTGS2, CD244, CD86, TLR4, CD1C, PPBP, CX3CR1,* and *CD200R1* (Figure 4B). *CD86* and *TLR4* were the ‘hub-genes’, each with 6 edges connected to 6 nodes. *CD200R1* was the top down-regulated gene by −3.7-fold. This network of down-regulated genes demonstrated a connection between monocyte activation genes *CD86, TLR4,* and *CD244*, and the gene associated with monocyte migration, *CX3CR1*.

Within all 75 GOIs, STRING identified the largest network of 26 genes (Figure 4C). The ‘hub-genes’ with the most connections included *TLR4* with 10 edges and *CD86* with 9 edges. *CD7* was the most up-regulated gene, and *CD200R1* the most down-regulated.

### 2.3. Functional Phenotyping of CD163+ Monocytes

The expression of cell surface markers representing various monocyte functions was quantified as a subset of the CD163+ monocytes (Table 2). The proportion of chemokine receptor CCR5+ cells was 1.3-fold lower, and CD11b+, CD11c+, and CD31+, indicators of cell adhesion and trans-endothelial migration were reduced 4.5, 2.2, and 8.7-fold, respectively, in CD163+ cells in D^+Comps^ compared to D^−Comps^ (all *p* < 0.05). Additionally, CD39 and CD86, which can promote immune regulation, were significantly decreased in D^+Comps^ (3.1- and 3.9-fold, respectively, both *p* < 0.05). Interestingly, the positive proportion of the toll-like receptors CD282 and CD284 and activation marker CD38 were very low in CD163+ cells in both groups, with no difference between them. The marker involved in phagocytosis and clearance, CD68, was highly expressed in CD163+ cells in both groups and was slightly lower in D^+Comps^ compared to D^−Comps^. 

### 2.4. Gene–Protein Interaction Network

**Figure 5:**
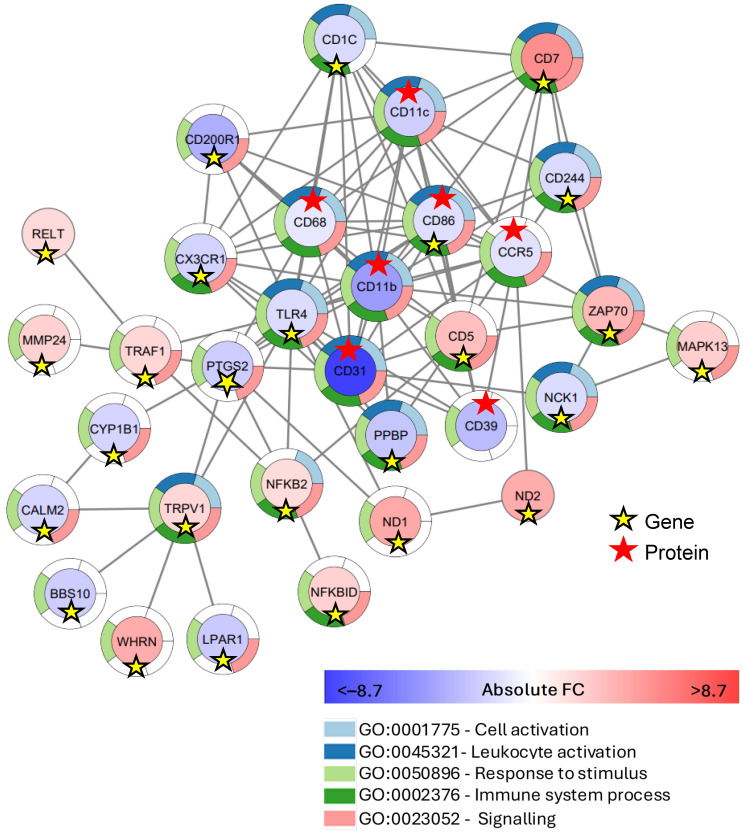
Gene–protein interactions network illustrating the largest connected network of ‘genes of interest’, combined with significantly different cell surface markers in CD163+ monocytes between individuals with long-term diabetes and complications (D^+Comps^) and those without complications (D^−Comps^). Genes were considered significantly down- or up-regulated if the fold change was ≥1.5 (BHp < 0.05). Proteins were considered significantly different at *p* < 0.05. The color of the circular node indicates the intensity of the fold change, confirmed by the legend below. The sectioned multicolor outline of each node indicates the involvement of respective genes, with the top five most enriched GO categories including biological processes. Yellow star indicates genes, whereas red star indicates protein.

A gene–protein interaction network was created, combining the 75 GOIs with the significantly different cell surface markers including CCR5, CD11b, CD11c, CD31, CD39, CD86, and CD68. This analysis revealed a network of 32 nodes in which CD11b and *TLR4* were the ‘hub-nodes’ with 16 edges, each connected to 16 nodes (Figure 5). CD86, with 14 edges connected to 14 nodes, was found to be down-regulated at both the gene and cell surface levels (Figure 5). The ‘hub-node’ CD11b was connected to genes including *TLR4*, *PPBP, PTGS2,* and *ZAP70,* as well as various cell surface markers. *TLR4* was linked to genes *TRPV1, NFKB2, TRAF1, CD200R1, CX3CR1,* and *PPBP,* and surface markers CD86, CD68, CD31, and CD39 (Figure 5). CD86 was in connection with genes *CD244, PTGS2,* and *ZAP70,* and cell surface markers CD31 and CD39. 

*CD7* was the most up-regulated by 5.1-fold and was connected to 8 nodes, including proteins CD11c, CD11b, CD68, and CCR5, and genes *CD1C, CD5*, *CD244,* and *ZAP70*. CD31 was most down-regulated by −8.7-fold and was connected to 12 nodes, including genes *MMP24*, *PTGS2, CD1c, CD5,* and *NK1*. There were two notable low-density ‘hub-genes’ within this network including *TRPV1,* connected to *TLR4, CALM2, BBS10, WHRN,* and *LPRN* and the pro-inflammatory gene *NFKB2,* connected to *TLR4, TRAF1*, *PTGS2,* and *CD5* (Figure 5).

## 3. Discussion

The global rise of diabetes and its accompanying, progressive micro- and macrovascular complications warrant the need for therapeutic measures which can alleviate or halt the progression of complications, resulting in increased capacity to manage diabetes. Chronic low-grade inflammation is a common feature found in people with diabetes, and may be a driver for the progressive nature of diabetes complications [4]. 

**Figure 6:**
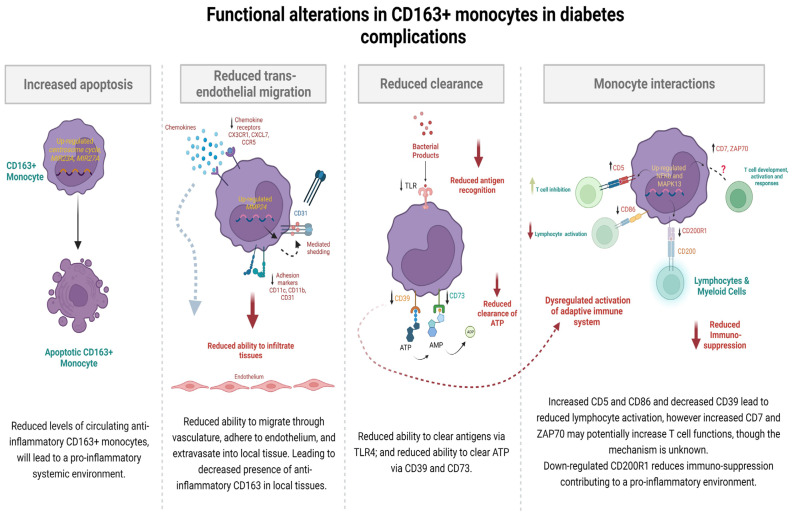
An infographic depicting the RNA and protein level functional alterations in the CD163+ monocytes of individuals with diabetes-related complications. The first panel describes mechanisms of increased apoptosis, the second panel depicts mechanisms involved in reduced trans-endothelial migration, the third panel depicts reduced CD163+ monocyte function, whilst the fourth panel depicts monocyte interactions.

The anti-inflammatory myeloid scavenger receptor CD163 has been reported to be decreased in people with diabetes complications [5]. Using RNA-seq and mass cytometry, we observed that both RNA and phenotypic profiles of circulating CD163+ monocytes were different in those with diabetes and complications compared to those without complications. In the context of complications, we reported enriched centrosome cycle genes and up-regulated miRNAs implicated in apoptosis, suggesting a decreased number of anti-inflammatory CD163+ monocytes (Figure 6). Additionally, in individuals with diabetes-related complications, CD163+ monocytes exhibit reduced abilities for cell recruitment and trans-endothelial migration, along with dysregulated activation of their adaptive immune system (Figure 6). These findings suggest that the anti-inflammatory nature and functionality of the CD163+ monocytes are diminished in cases of diabetes complications, and this may reduce the potential to resolve inflammation.

Deep RNA profiling of circulating CD163+ monocytes in our study revealed that in those with diabetes-related complications, the genes associated with the ‘regulation of centrosome cycle’ were up-regulated and enriched. Wang and colleagues reported enriched centrosome amplification within the peripheral blood mononuclear cells of individuals with type 2 diabetes, although the complication status of the participants was unknown [11]. The centrosome and its associated components determine the geometry of microtubule arrays throughout the cell cycle, which influence cell shape, polarity, motility, chromosome segregation, and cell division [12]. Dysregulation in the centrosome cycle can implicate cellular apoptosis [13], which would correspond with the up-regulated microRNAs *MIR23A* and *MIR27A* found in CD163+ cells in this study, as they also contribute to increased cellular apoptosis [14,15]. Our finding of potentially increased cellular apoptosis of circulating CD163+ monocytes would explain the previous finding of decreased circulating peripheral CD163+ monocytes in individuals with diabetes-related complications [5]. The reduced presence of anti-inflammatory CD163+ monocytes in the systemic circulation would support the notion of a prolonged inflammatory state in the systemic environment of individuals with diabetes-related complications (Figure 6).

A significantly distinct functional profile of CD163+ monocytes was observed in this study between individuals who have diabetes with and without complications. Specifically, genes and cell surface markers involved in trans-endothelial migration and the recruitment of CD163+ monocytes were significantly suppressed and the expression of chemokine receptors *CX3CR1*, *CXCL7 (PPBP),* and CCR5 were down-regulated in those with diabetes complications. When coupled with the decreased proportion of the adhesion markers CD11b, CD11c, and CD31, this would be indicative of the reduced ability of CD163+ monocytes to adhere to the endothelium of vasculature and extravasate into local tissues (Figure 6). Furthermore, the association between the up-regulated gene *MMP24* and the down-regulated protein CD31, as discovered by the interaction network analysis, suggests MMP24 may be involved in reducing the cell surface expression of CD31, possibly through mediating ectodomain shedding, based on previous findings reported in animal studies of retinopathy in diabetes [16]. 

In addition, the function of antigen recognition by the CD163+ monocyte was decreased in individuals with diabetes and complications compared to those without, although a very low proportion of TLR4+ cells was detected in the CD163+ cells from both groups. The down-regulated *TLR4* would be consistent with a diminished capacity of the CD163+ monocytes to recognize and internalize antigens such as pathogen-associated molecular pattern molecules (PAMPs) and damage associated molecular pattern molecules (DAMPS), leading to an inadequate immune response, impaired pathogen clearance [17], and continued inflammation (Figure 6). The down-regulated *TLR4* in CD163+ cells, which are a ‘hub-node’, raises the proposition that the reduced TLR4 functionality would trigger a cascade of events involving both monocyte function and immune response, thereby contributing to the development of complications [18]. Contrastingly, an earlier study in a rat model of retinopathy in diabetes reported that increased TLR4 in retinal extracts was associated with disease progression [19] and that the blockade of the TLR4 signaling pathway reduced inflammation [19,20,21], thus indicating that the TLR4 signaling must be tightly regulated to prevent excessive inflammation.

The ability of the CD163+ monocytes to clear and regulate extracellular ATP may be potentially diminished in individuals with diabetes complications due to the decreased expression of the ectonucleotides CD39 and CD73 (Figure 6). Dysregulation of extracellular ATP may impair the supply and formation of adenosine, an anti-inflammatory mediator, which would lead to a pro-inflammatory environment [22]. Similarly, a recent study found the lowest CD39 expression in circulating lymphocytes in individuals with diabetes-related complications, compared to those without [23]. Decreased CD39 expression has also been implicated in reduced monocyte-mediated lymphocyte activation [24]. This suggests that the unresolving nature of diabetes-related complications may be due to the dysregulated interaction between the antigen-presenting CD163+ monocytes and the adaptive immune system (Figure 6).

We observed an increased expression of the gene *CD5* in CD163+ monocytes in individuals with diabetes-related complications. *CD5* has been reported to suppress the activation of peripheral T cells through the inhibition of T cell receptors at the immunological synapse [25]. Similarly, CD86, a co-stimulatory molecule necessary for effective T cell activation and survival [26] was also down-regulated in the CD163+ monocytes in D^+Comps^. As summarized in Figure 6, our results suggest that CD163+ monocyte-mediated T cell activation was down-regulated in individuals with diabetes-related complications, due to the increased *CD5* and decreased CD86 expression. However, we also propose an alternative pathway for T cell activation involving the up-regulated expressions of *CD7* and *ZAP-70,* which are both implicated in the initiation of T cell responses via the antigen receptor [27,28]. This may indicate a possible connection point between the non-specific immune response driven by monocytes and the adaptive and specific immune response mediated by T cells.

Furthermore, in diabetes-related complications, CD163+ monocyte-mediated immunosuppression was potentially reduced due to the decreased expression of the immune inhibitory receptor *CD200R1* (Figure 6). The interaction of *CD200* with its receptor *CD200R1* typically evokes an immunosuppressive response [29]. Our study therefore supports the concept that reduced immunosuppression leads to prolonged inflammation at the organ sites, ultimately resulting in a long duration of diabetes with complications. Interestingly, in our study, *CD200R1* was connected to the down-regulated monocyte function markers CD86 and *TLR4* (Figure 4B), implicating that the *CD200-CD200R1* immunosuppressive pathway is responsible for the decreased monocyte-mediated T cell activation (Figure 6).

In this study, a satellite network identified the up-regulated *TRPV1* gene in individuals with diabetes-related complications. Earlier studies have coherently reported that blockade of *TRPV1* in rodents with type 2 diabetes halted disease progression [30], thus indicating that a *TRPV1* antagonist may be a therapeutic target for the disease progression of diabetes by improving glucose intolerance and correcting dysfunctional insulin secretion [30]. 

A limitation of the current study is the small sample size. Additionally, despite generating extensive data on various aspects of cellular functions, we restricted the differential gene analysis to areas of particular interest for this study. While we have comprehensively profiled the RNA transcriptome and surface marker phenotype of CD163+ monocytes in the context of diabetes complications, further research is needed to uncover the causative mechanisms underlying this distinct cell profile. Future studies will focus on identifying the genomic mechanisms involved in lipid, insulin, or glucose dysregulation in CD163+ monocytes. Furthermore, we will employ the weighted gene co-expression network analysis to thoroughly explore the dataset and expand the sample size to facilitate a more comprehensive investigation. 

In conclusion, this study is the first to comprehensively characterize the RNA profile and phenotype of CD163+ monocytes in individuals with long-term diabetes and complications, presenting new implications for the role of CD163+ monocytes in diabetes-related complications. It identified specific enriched genes in CD163+ monocytes that are involved in centrosome cycle regulation, and miRNAs that are associated with cell death, consistent with the decreased CD163+ cells. Additionally, decreased expression of genes and cell surface markers involved in monocyte activation, recruitment, and immune regulation was observed. These results suggest that CD163+ monocytes have unique functions in diabetes-related complications, and it is their loss of function and programmed death that leads to the increased and persistent tissue complications in diabetes. Prospective research is now needed to build upon our data and define the role of these cells in the development of complications in diabetes. 

## 4. Materials and Methods

### 4.1. Study Design

Adults with a diabetes duration greater than 10 years, either with (D^+Comps^; *n* = 6) or without (D^−Comps^; *n* = 6) any microvascular or macrovascular complications, were recruited from the Diabetes Centre, Royal Prince Alfred Hospital, Sydney, Australia for this study. The sample size for each group was determined based on our previous studies on CD163 monocytes. A power analysis was conducted, considering a significance level (alpha) of 0.05 and a desired power of 80% (ClinCalc LLC). The analysis indicated that a minimum of five participants per group would be required to achieve statistical significance. To account for potential sample loss during the experiment, the final sample size was adjusted to six participants per group. 

Complication status was assessed as described previously [31]. Briefly, individuals were included based on the presence of retinopathy, which was confirmed by fundal examination and/or photography. The absence of nephropathy was confirmed by normal serum creatinine and a urinary albumin–creatinine ratio < 2.5 mg/mmol for men and < 3.5 mg/mmol for women. Participants were considered to have macrovascular disease if they had any relevant symptoms of vascular disease or had a reported history of abnormal investigation or prior macrovascular event. This study has been approved by the Human Ethics Review Committee of Sydney Local Health District (Protocol No. X14-0074), and was carried out in accordance with the principles of the Declaration of Helsinki as revised in 2000. All participants gave their written informed consent.

### 4.2. RNA Sequencing of CD163+ Monocytes

#### 4.2.1. Isolation of CD163+ Cells

CD163+ monocytes were isolated from freshly collected blood by fluorescence-activated cell sorting FACSAria (Becton Dickinson, San Jose, CA, USA), using the antibodies CD45-PerCP-Cy5.5 (Becton Dickinson, San Jose, CA, USA), CD14-PE-Cy7 (Becton Dickinson, San Jose, CA, USA), and CD163-APC (R&D Systems, Minneapolis, MN, USA). The dead cells were excluded by DAPI (Invitrogen, Waltham, MA, USA) staining. The purity of the isolated CD163+ cells approached 95%, shown as a representative image in Supplementary Figure S1.

#### 4.2.2. RNA Extraction and Sequencing

Total RNA was extracted from the isolated CD163+ monocytes by RNeasy Micro Kit (QIAGEN, Hilden, Germany) according to the manufacturer’s protocols. The quality and purity of the RNA were determined by the optical density on a NanoDrop 2000 (Thermo Fisher Scientific Inc., Waltham, MA, USA) and Agilent Bioanalyzer RNA Nano Chip Bioanalyzer (Agilent Technologies, Santa Clara, CA, USA). Illumina Stranded Total RNA Prep (Illumina, San Diego, CA, USA) was used to convert RNA into sequencing-ready libraries and the Illumina Ribo-Zero Plus rRNA Depletion Kit (Illumina, San Diego, CA, USA) was used to remove abundant RNA by enzymatic depletion. RNA from each of the 12 samples was normalized, then progressed to generate the sequence data using NovaSeq 6000 System (Illumina, San Diego, CA, USA), with the resultant FASTQ files used for data analysis. RNA-seq was performed by the Australian Genome Research Facility (Melbourne, VIC, Australia).

#### 4.2.3. Quality Control and Differential Gene Expression Analysis

The RNA-seq data were analyzed using the Galaxy web platform at *usegalaxy.org* [32]. An initial multi-dimensional scaling (MDS) plot was generated to visualize the degree of separation between D^+Comps^ and D^−Comps^ [33] (Supplementary Figure S2). FASTQ files were generated after a quality control assessment using the ‘FastQC’ tool [34], and the analysis followed the pipeline shown in Supplementary Figure S3. The raw reads were trimmed using ‘Trimmomatic’ to remove the adaptors [35], re-assessed using FastQC, and aligned individually to the human reference genome Hg38 using ‘HISAT2’ (version 2.2.1) [36]. The mapped reads were then quantified using ‘feature counts’, wherein genes were normalized and those less than 1 count per million were filtered out. DEGs between D^+Comps^ and D^−Comps^ were analyzed using Limma-Voom’ (version 3.50.1), with the following criteria: log_2_ fold change and an adjusted *p*-value < 0.05 using the Benjamini–Hochberg method [37,38]. The list of DEGs was then sorted to identify up- or down-regulated genes using a cut-off value of ≥ 1.5 absolute FC (BHp < 0.05), as described by Lurier et al. [39]. The list of GOIs was generated based on the genes with an FC ≥ 1.5 using three different screening procedures, as shown in Figure 1. Firstly, the top 20 most significantly up- and down-regulated genes were selected. Secondly, the Panther 17.0 classification system was used to evaluate the genes with an FC ≥ 1.5 using the over-representation test and functional classification of biological processes (Figure 2) [40,41]. The genes involved in diabetes, inflammation, immune system functions, or monocyte functions including biological adhesion, immune system processes, localization, and response to stimulus and signaling were included in the GOIs. Finally, the DEGS with an FC ≥ 1.5 were manually searched and added to the GOIs list if their role related to diabetes, monocyte function, inflammation, cytokines, chemokines, or MMPs. 

In order to identify the interactions between individual GOIs, a PPIs network was established using the STRING database and the network was visualized by Cytoscape (version 3.9.1) [42,43]. Nodes in the PPIs network represent genes, while the edges represent interactions between the genes. In this study, nodes with the greatest number of edges in connection with others were termed ‘hub’ genes.

GO and KEGG pathway enrichment analysis were conducted individually on the up- and down-regulated GOIs, as well as the combined GOIs list. The top five most significant enriched ‘GO’ categories were reported (FDR < 0.05).

### 4.3. Functional Phenotyping by Mass Cytometry

#### 4.3.1. Cell Preparation and Staining with Antibodies for Mass Cytometry

Blood samples were stored in a proteomic stabiliser and the erythrocytes were lysed using the Thaw-Lyse kit as per the manufacturer’s protocol (Smart Tube Inc., Las Vegas, NV, USA). After lysis, a total of 2 × 10^6^ white blood cells were blocked with heparin to reduce non-specific eosinophil staining [44]. Samples were then incubated with anti-CD45 antibody as ^106^Pd-CD45 for D^+Comps^ and ^108^Pd-CD45 for D^−Comps^ samples. Paired D^+Comps^ and D^−Comps^ samples were then stained with cell surface markers, as shown in Supplementary Table S3. DNA intercalator iridium in a perm buffer with paraformaldehyde was used to stain DNA in the cells. 

Subsequently, cells were counted at approximately 1 × 10^6^ cells/mL with EQ calibration beads and analyzed using a time-of-flight mass cytometer (Helios™, CyTOF^®^system, Fluidigm, San Francisco, CA, USA).

#### 4.3.2. Gating Strategy Analysis of CD163+ Monocyte Profile

Mass cytometry data were analyzed with FlowJo (FlowJo LLC., Ashland, OR, USA). The gating strategy used to profile the CD163+ monocytes is shown in Supplementary Figure S4. Briefly, normalization beads were excluded, then cell singlets were gated on event length against DNA. The paired D^+Comps^ and D^−Comps^ were de-barcoded as individual samples and negative selection was sequentially applied to exclude platelets, granulocytes, T-cells, B-cells, and NK cells, then positive selection was applied using HLADR. Subsequently, CD163+ monocytes were gated under CD14+ monocytes. Finally, the profile of the CD163+ monocytes was analyzed within the CD163+ monocyte population using the cell surface markers to identify various cell functions. The results were reported as a percentage of the CD163+ monocytes. These studies were undertaken in a blinded manner to the group source of the CD163+ monocytes (complications present or absent).

### 4.4. Gene–Protein Combined PPIs Network

To investigate the interaction between genes and cellular surface markers, the significantly different cell surface markers across D^+Comps^ and D^−Comps^ were included in the GOIs list, and the PPIs network was analyzed as described above. The nodes with the greatest number of edges in connection with others were termed ‘hub node(s)’.

### 4.5. Statistical Analysis

Data analysis of clinical characteristics and the immunophenotyping of CD163+ monocytes was performed using GraphPad Prism 9 (GraphPad Software 9.5.0 (730), San Diego, CA, USA). Continuous data were assessed for normality and presented as mean ± SD for parametric data, and median, IQR, or proportions for non-parametric data, as indicated. Grouped data were compared with either Welch’s t-test for parametric data, Mann–Whitney test for non-parametric data, or Fisher’s exact test to identify the differences between groups.

For RNA-seq data, the DEGs were accepted as significant when the adjusted *p*-value BHp was <0.05. The over-representation test identified significantly enriched biological processes, as determined using Fisher’s exact test (*p* < 0.05) with FDR correction. Finally, the PPIs network utilized an FDR < 0.05 to identify enriched GO categories and KEGG pathways.

## Figures and Tables

**Table 1:** Clinical characteristics of individuals with diabetes with and without complications.

	D^+Comps^ (*n* = 6)	D^−Comps^ (*n* = 6)	*p*-Value
Age (years)	67.5 ± 18.3	73.2 ± 5.2	ns
Men/women (*n*)	4/2	3/3	ns
Duration of diabetes (years)	26.9 ± 5.3	22.0 ± 5.9	ns
BMI (kg/m^2^)	26.8 ± 4.0	30.5 ± 5.9	ns
HbA_1c_ (mmol/mol)	64 (55–66)	61 (40–64)	ns
HbA_1c_ (%NGSP units)	8.0 (7.2–8.2)	7.7 (5.8–8.0)	ns
Total cholesterol (mmol/L)	3.7 ± 0.6	4.3 ± 1.0	ns
HDL-c (mmol/L)	1.4 ± 0.4	1.4 ± 0.1	ns
LDL-c (mmol/L)	1.7 ± 0.5	2.3 ± 0.9	ns
Triglycerides (mmol/L)	1.4 ± 0.2	1.6 ± 0.8	ns
Serum creatinine (µmol/L)	142.0 (83.8–165.8)	67.5 (48.6–75.5)	ns
U Alb (mg/24 hrs)	36 (19–212)	8.6 (5.1–14.15)	ns
UACR (mg/mol)	4 (2.1–12.5)	1.2 (0.8–5.6)	ns
eGFR (mL/min/1.73 m^2^)	59.8 (40.1–97.6)	84 (66.3–91.3)	ns
Systolic BP (mmHg)	130 (121–143)	134 (120–146)	ns
Diastolic BP (mmHg)	64 ± 7	76 ± 9	0.03
Medications			
Statin (*n*)	5	5	ns
Aspirin (*n*)	2	3	ns
Insulin (*n*)	5	1	0.01
Glucose-lowering therapy (*n*)	2	6	ns

Results are expressed as mean ± SD or median with interquartile range (IQR) or proportion. Significance was accepted at *p* < 0.05 and no significance indicated by ns. U Alb, urinary albumin; UACR, urinary albumin/creatinine ratio; eGFR, estimated glomerular filtration rate; BP, blood pressure.

**Table 2:** Functional phenotyping of CD163+ monocytes in individuals with diabetes with or without complications.

Function	Positive % of CD163+ Cells	D^+Comps^ (*n* = 6)	D^−Comps^ (*n* = 6)	Fold Change	*p* Value
Activation	CD282 (TLR2)	0.4 (0.1–3.7)	0.45 (0.2–0.8)	−0.1	ns
	CD284 (TLR4)	0.3 (0.2–3.6)	0.5 (0.3–0.7)	−1.9	ns
	CD38	0.4 (0.2–3.1)	2.4 (1.7–4.1)	−6.0	*p* = 0.06
Chemokine receptors	CD192 (CCR2)	2.6 (1.7–4.8)	0.9 (0.5–3.6)	2.6	ns
	CD195 (CCR5)	59.6 (45.9–70.9)	78.4 (72.9–78.7)	−1.3	*p* < 0.05
	CX3CR1	0.8 (0.7–2.1)	0.8 (0.4–0.9)	−1.1	ns
	CXCR3	9.6 ± 7.3	8.2 ± 9.6	0.8	ns
Adhesion and migration	CD11b	12.1 (7.8–22.2)	54.4 (29.9–58.6)	−4.5	*p* < 0.05
	CD11c	27.8 ± 11.5	60.1 ± 22.3	−2.2	*p* < 0.05
	CD31 (PECAM-1)	4.7 (3.5–13.9)	41.0 (18.8–52.3)	−8.7	*p* < 0.05
Immune regulation	CD39	4.2 ± 2.4	13.0 ± 6.8	−3.1	*p* < 0.05
	CD73	0.7 (0.5–3.6)	1.3 (0.9–2.1)	−1.9	*p* = 0.06
	CD80	1.8 (1.3–3.1)	1.3 (0.8–1.6)	0.7	ns
	CD86	3.2 ± 1.1	12.5 ± 7.2	−3.9	*p* < 0.05
	CD206	0.1 (0.05–1.05)	0.25 (0.03–1.9)	−2.8	ns
Phagocytosis and clearance	CD36	99.2 (82.2–99.6)	92.0 (65.7–99.5)	1.1	ns
	CD68	97.3 (93.7–98.2)	98.8 (98.1–99.7)	−1.0	*p* < 0.05

Results are expressed as mean ± SD or median with interquartile range (IQR) or proportion. Folder changes were expressed as D^+Comps^ compared to D^−Comps^ and significance accepted at *p* < 0.05. No significance indicated by ns.

## Data Availability

All group data have been included in this article. The datasets for individuals are not publicly available due to ethical restrictions.

## Supplementary Materials

The following supporting information can be downloaded at: https://www.mdpi.com/article/10.3390/ijms251810094/s1.
