# Supplementary material for: Deep Immune and RNA Profiling Revealed Distinct Circulating CD163+ Monocytes in Diabetes-Related Complications
Source: Int J Mol Sci. 2024 Sep 19;25(18):10094. doi: 10.3390/ijms251810094 (PMC11432403; doi:10.3390/ijms251810094)
Supplement: Supplementary file 1 [file ijms-25-10094-s001.zip › ijms-3168047-supplementary.pdf]

Supplementary Figure S1

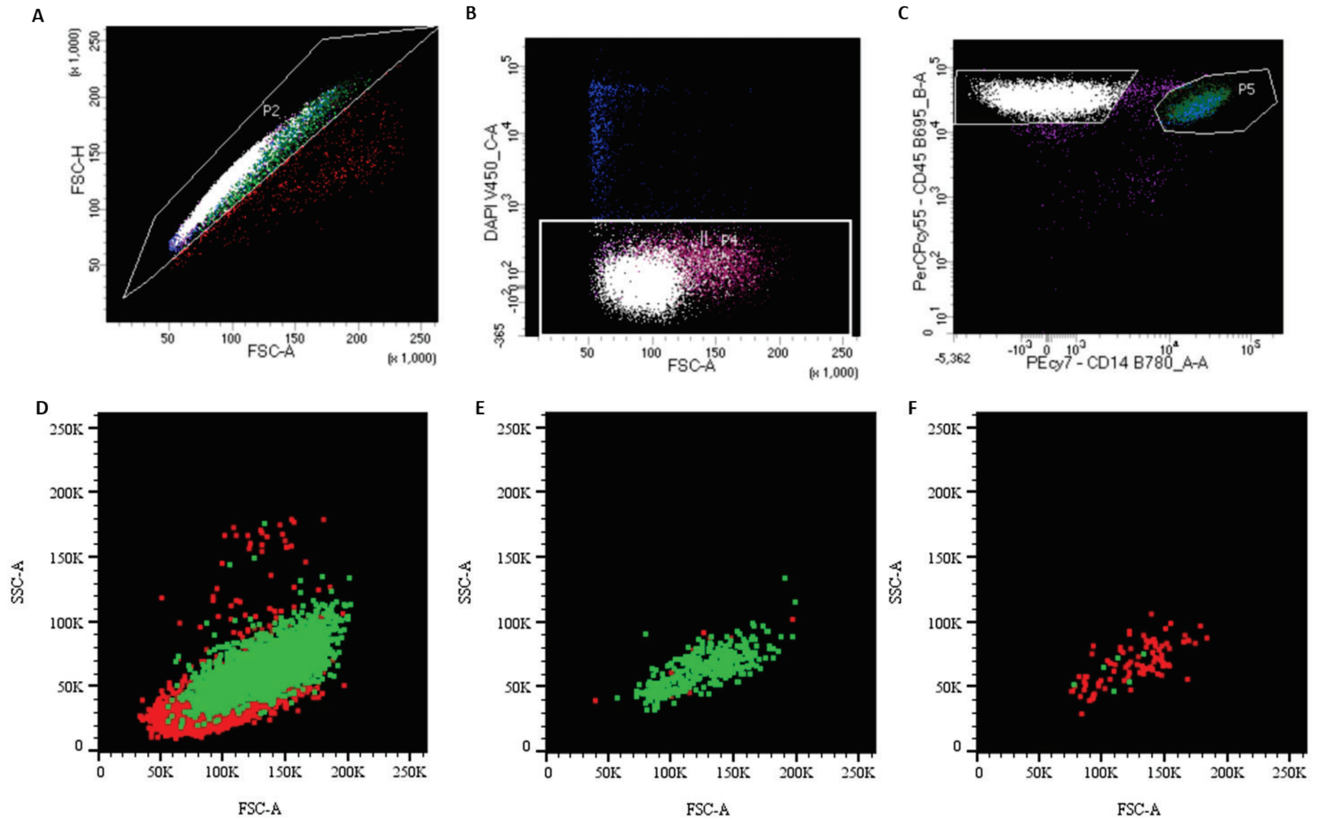

Subset Name	Freq. of Parent
CD163+	22.3
CD163-	76.5

Blood before sorting

Subset Name	Freq. of Parent
CD163+	94.6
CD163-	4.27

CD163+ monocytes

Subset Name	Freq. of Parent
CD163+	6.32
CD163-	90.5

CD163- monocytes

Figure S1. Isolating and detecting the purity of the CD163+ monocytes from blood by flow cytometry. (A-C) Gate strategies to isolate CD163+ monocytes: (A) Singlet selection (B) Identification of live cells as DAPI negative, and (C) Isolation of CD163 cells under Gate P5, CD45+CD14+ monocytes. The proportion of CD163+ and CD163- monocytes were detected: (D) before cell sorting, and (E & F) after sorting from (E) the isolated CD163+ cells (F) the isolated CD163- cells. Green dots indicate CD163+ cells and red dots indicate CD163- cells.

Supplementary Figure S2

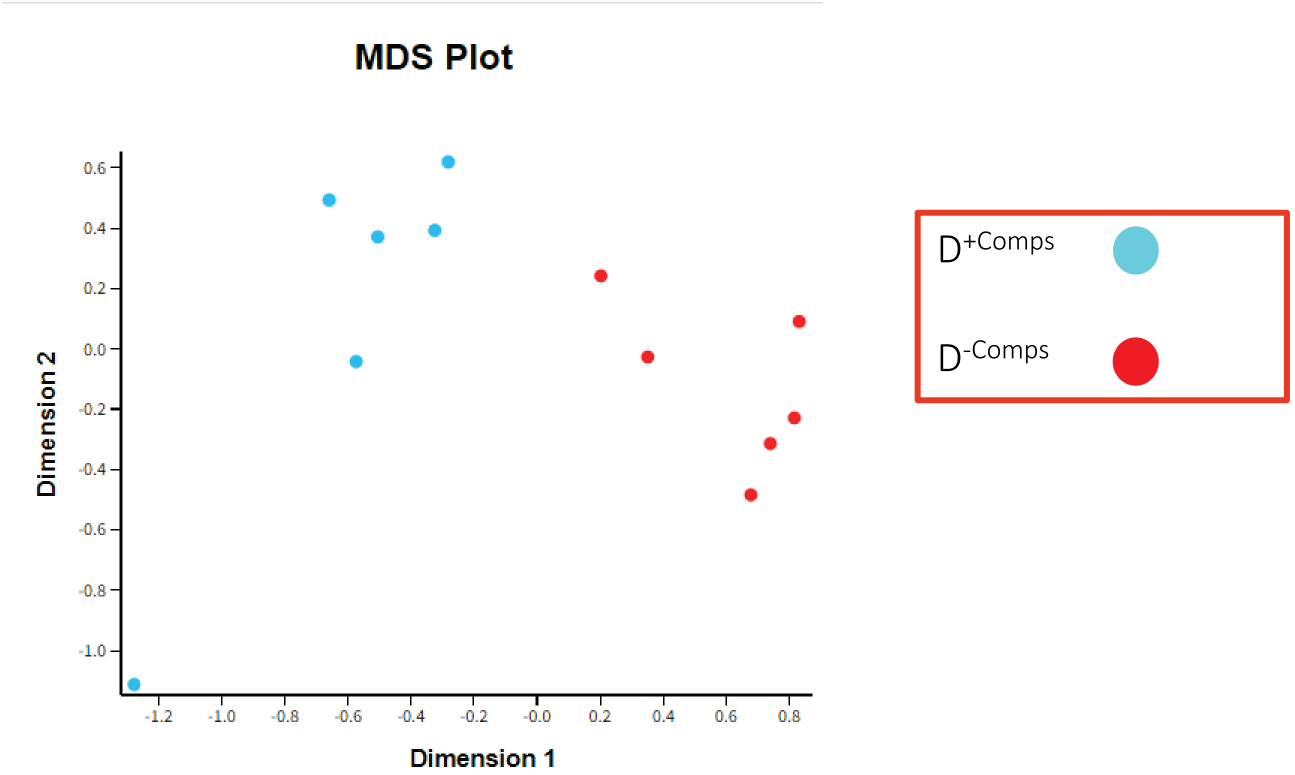

Figure S2. A multi-dimensional scaling (MDS) plot of RNA-seq data showing variation among samples. Each coloured point represents the individual sample belonging to either D^{+Comps} (blue dot) or D^{-Comps} (red dot). The distance between each pair of points correlates to the dissimilarity between the two samples.

Supplementary Figure S3

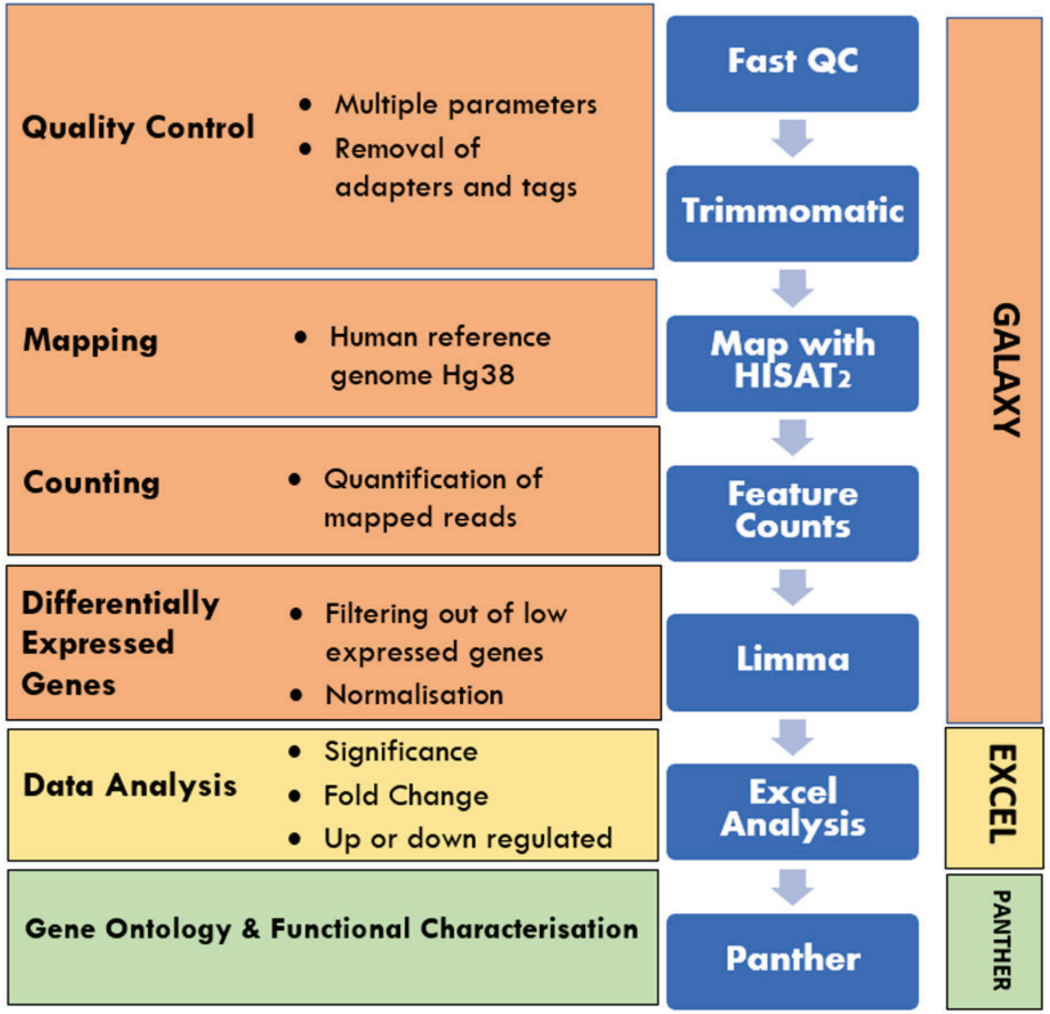

Figure S3. The RNA-sequencing analysis pipeline to identify differentially expressed genes, gene ontology and functional characterization in CD163+ monocytes in D^{+Comps} and D^{-Comps} using Galaxy and PANTHER classification tools, is shown.

Supplementary Figure S4

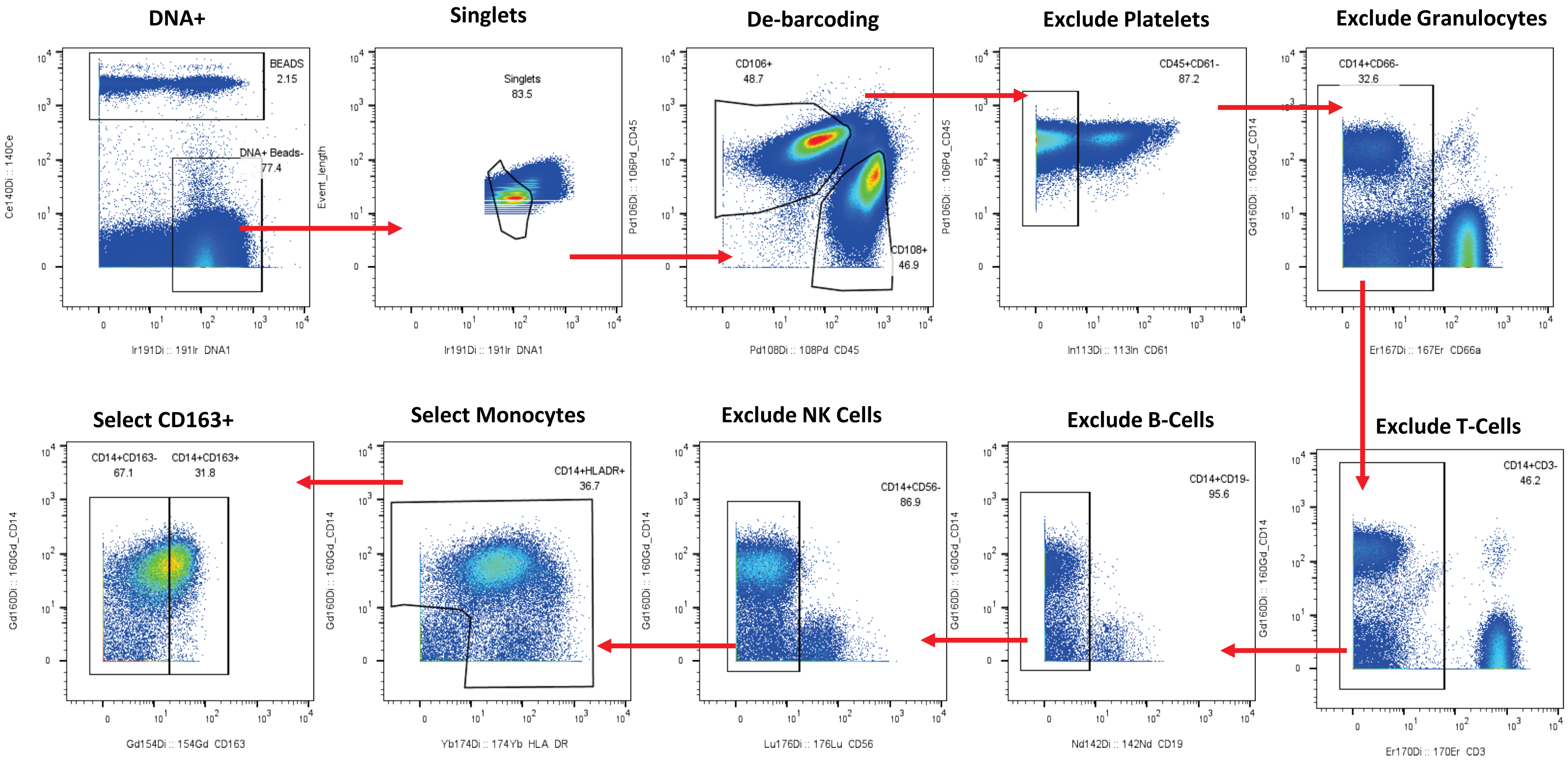

Figure S4. The gating strategy established to target the CD163+ monocytes is shown using a negative selection to exclude platelets, granulocytes and lymphocytes, from CD45+ white blood cells.

Supplementary Table S1

The complication status of individual participants with diabetes.

Study Code	Age (years)	Duration of Diabetes (years)	Macrovascular	Microvascular	
				Nephropathy	Retinopathy and Grading
**Ext 4**	70	23	**-**	+	+/1
**Ext 5**	39	25	+	+	+/1
**Ext 6**	56	31	**-**	**-**	+/1
**Ext 8**	87	21	**-**	**-**	+/5
**Ext 10**	67	26	+	+	+/2
**Ext 13**	86	35	+	+	+/3
**Ext 1**	66	14	**-**	**-**	**-**
**Ext 2**	80	29	**-**	**-**	**-**
**Ext 12**	69	28	**-**	**-**	**-**
**Ext 19**	72	21	**-**	**-**	**-**
**Ext 20**	76	20	**-**	**-**	**-**
**Ext 21**	76	19	**-**	**-**	**-**

Retinopathy Worst Ever Grading: 0 = Nil ; 1 = Minimal ; 2 = Mild/Mod NPDR;
3 = Mod/Severe NPDR; 4 = Proliferative; 5 = Macula Oedema.

Supplementary Table S2

List of the 'Genes of Interest' including the up and down-regulated genes (≥1.5 FC; BHp<0.05).

**Gene Symbol**	**Absolute FC**	**Adjusted *P* value**	**Gene Function**
MIR23A*	6.6	0.002	MicroRNAs
MIR3648-1*	6.4	0.041	MicroRNAs
MIR27A*	6.3	0.003	MicroRNAs
CD7*	5.1	0.001	T-cell interactions and also in T-cell/B-cell interaction during early lymphoid development
PLEKHG2*	5.0	0.002	Regulates lymphocyte chemotaxis
LOC100129917*	4.7	0.005	-
MIR612*	4.6	0.002	MicroRNAs
TMEM184A*	4.3	0.008	Heparin receptor and mediates anti-inflammatory responses
ZSWIM4*	4.3	0.001	Predicted to enable zinc ion binding activity
LOC105371789*	4.2	0.004	An RNA Gene, and is affiliated with the lncRNA class.
TCTEX1D4*	4.1	0.002	Involved in microtubule-based movement
MIR23AHG*	4.0	0.001	An RNA Gene, and is affiliated with the lncRNA class
TRI-TAT1-1*	3.9	0.005	An RNA Gene, and is affiliated with the tRNA class
ND1*	3.9	0.026	Enables NADH dehydrogenase activity. Involved in mitochondrial electron transport.
ND2*	3.8	0.042	Enables NADH dehydrogenase activity. Involved in mitochondrial electron transport.
WHRN*	3.8	0.004	Organization and stabilization of sterocilia elongation and actin cystoskeletal assembly

ZBTB16*	3.8	0.010	Involved in cell cycle progression
BTBD19*	3.8	0.002	-
LOC105378949*	3.7	0.002	-
CTBP1-AS*	3.6	0.002	An RNA Gene, and is affiliated with the lncRNA class
MAPK8IP1Φ	3.4	0.005	Regulator of the pancreatic beta-cell function, susceptibility gene for type 2 diabetes
ZAP70†	3.2	0.001	Plays a role in T-cell development and lymphocyte activation
LOC286059Φ	3.1	0.006	TNF receptor superfamily member pseudogene
CD5†	2.9	0.004	Act as a receptor to regulate T-cell proliferation
NLGN3†	2.5	0.004	Neuronal surface protein
AMIGO3Φ	2.4	0.002	Cell adhesion
MAPK8IP3†	2.3	0.002	Regulate the activity of numerous protein kinases of the JNK signaling pathway
MAPK13†	2.2	0.011	Integration point for biochemical signals, and processes such as proliferation, differentiation, transcription regulation and development
APOA2Φ	2.2	0.006	High density lipoprotein particles
ADGRL1Φ	2.2	0.005	Cell adhesion and signal transduction
MMP24Φ	2.1	0.004	Breakdown of extracellular matrix in embryonic development, reproduction, and tissue remodeling
NFKBIDΦ	2.1	0.014	Enable NF-kappaB binding activity, T cell signaling and functions and regulation of gene expression
IL11RA†	1.9	0.028	Encodes the IL-11 receptor, a member of the hematopoietic cytokine receptor family
C1QTNF7-AS1Φ	1.9	0.024	Identical protein binding activity, collagen trimmer
ZBTB17†	1.9	0.007	Zinc finger protein involved in the regulation of c-myc.

TRAF1†	1.8	0.023	Mediates Signal transduction
ADGRB1Φ	1.8	0.048	Cell adhesion
TRPV1†	1.8	0.015	Detection and regulation of body temperature and nociception, mediates detection of noxious environmental stimuli
SMAGPΦ	1.7	0.048	Cell adhesion
ZBTB25†	1.7	0.007	Regulation of transcription by RNA polymerase II
FASNΦ	1.7	0.029	Catalyse synthesis of long chain saturated fatty acids
RELTΦ	1.7	0.014	Activates NF-kappaB pathway, selectively binds TRAF1, stimulates T cell proliferation
NFKB2†Φ	1.6	0.033	Functions as a central activator of genes involved in inflammation and immune function.
IRS2†	1.5	0.029	Mediates effects of insulin, insulin-like growth factor 1, and other cytokines
TRAF3IP2-AS1†	1.5	0.017	RNA Gene, and is affiliated with the lncRNA class.
TRAF4Φ	1.5	0.047	Mediates signal transduction
EMB†	-1.5	0.042	Involved in cell growth and development by mediating interactions between the cell and extracellular matrix
CD86†	-1.5	0.031	Pathogen recognition and activation of innate immunity
TLR4†	-1.6	0.027	Pathogen recognition and activation of innate immunity
NCK1†	-1.6	0.046	Signaling and transforming proteins
CD244†	-1.7	0.006	Expressed on NK cells and some T cells that mediate non-major histocompatibility complex (MHC) restricted killing
CD1C†	-1.7	0.005	Mediate the presentation of primarily lipid and glycolipid antigens of self or microbial origin to T cells
PALLD†	-1.8	0.029	Organizing the actin cytoskeleton

DSC2†	-1.9	0.005	Cell-cell junctions
TGFA†	-1.9	0.008	Growth factor which activates a signaling pathway for cell proliferation, differentiation and development
TMEM106B*	-1.9	0.048	Involved in dendrite morphogenesis and lysosome localization
CALM2*	-1.9	0.027	Calcium binding protein that plays a role in signaling pathways, cell cycle progression and proliferation
CYP1B1*	-1.9	0.038	Catalyze many reactions involved in drug metabolism and synthesis of cholesterol, steroids and other lipids
LINC01094*	-1.9	0.028	RNA Gene, and is affiliated with the lncRNA class
GSKIP*	-2.0	0.020	Involved as a negative regulator of GSK3-beta in the Wnt signaling pathway
BTG2*	-2.0	0.006	Antiproliferative properties, involved in the regulation of the G1/S transition of the cell cycle
NEIL3*	-2.0	0.038	Initiate the first step in base excision repair by cleaving bases damaged by reactive oxygen species
ZNF772*	-2.0	0.049	Regulation of transcription by RNA polymerase II
CX3CR1*†	-2.0	0.016	Involved in the adhesion and migration of leukocytes
CENPQ*	-2.2	0.006	Involved in assembly of kinetochore proteins, mitotic progression and chromosome segregation.
DHRS9*	-2.2	0.039	Dehydrogenase/reductase
ZFP3*	-2.2	0.019	Regulation of transcription by RNA polymerase II
PRICKLE1*	-2.2	0.035	Negative regulator of the Wnt/beta-catenin signaling pathway
BBS10*	-2.3	0.025	Member of the Bardet-Biedl syndrome (BBS) gene family
ZIK1*	-2.3	0.003	Regulation of transcription by RNA polymerase II
PTGS2*	-2.3	0.042	It is responsible for the prostanoid biosynthesis involved in inflammation and mitogenesis.

LPAR1*	-2.4	0.006	Proliferation, platelet aggregation, smooth muscle contraction, chemotaxis, and tumor cell invasion
PPBP*†	-2.6	0.023	Potent chemoattractant and activator of neutrophils
STEAP4*	-3.0	0.043	Involved in adipocyte development and metabolism
CD200R1*	-3.7	0.014	The receptor-substrate interaction may function as a myeloid downregulatory signal

*Indicates the gene is from the 20 most up or down-regulated

† Indicates the gene is from selected biological processes

ɸ Indicates the gene is from manual search of original DEG list based on our interest

Supplementary Table S3

The list of immunophenotyping panel for mass cytometry used in this study including the target CD marker, it's conjugated rare-earth metal isotype, the company details of the antibodies, and purpose of the marker within this panel.

Metal		Clone	Company	Target	Marker purpose
^{106}Pd	Palladium	HI30	Biolegend	CD45	Lineage/Barcode
^{108}Pd	Palladium	HI30	Biolegend	CD45	Lineage/Barcode
^{113}In	Indium	VI-PL2	Biolegend	CD61	Lineage
^{167}Er	Erbium	YTH71.3	Abcam	CD66	Lineage
^{170}Er	Erbium	UCHT1	Biolegend	CD3	Lineage
142Nd	Neodymium	HIB19	Biolegend	CD19	Lineage
^{176}Yb	Ytterbium	REA196	Miltenyi	CD56	Lineage
^{160}Gd	Gadolinium	M5E2	BD	CD14	Lineage
^{174}Yb	Ytterbium	L243	Biolegend	HLA-DR	Lineage
^{154}Sm	Samarium	GHI/61	Biolegend	CD163	Anti-inflammatory
^{171}Yb	Ytterbium	TL2.1	Biolegend	CD282 (TLR2)	Activation
^{149}Sm	Samarium	HTA125	BD	CD284 (TLR4)	Activation
^{172}Yb	Ytterbium	HIT2	BD	CD38	Activation
^{151}Eu	Europium	K036C2	Biolegend	CD192(CCR2)	Chemokine receptor
^{144}Nd	Neodymium	HEK/1/85a	Bio-Rad	CD195(CCR5)	Chemokine receptor
^{164}Dy	Dysprosium	2A9-1	Biolegend	CX3CR1	Chemokine receptor

^{163}Dy	Dysprosium	REA232	Miltenyi	CXCR3	Chemokine receptor
^{209}Bi	Bismuth	ICRF44	Biolegend	CD11b	Adhesion
^{115}In	Indium	Bu15	Biolegend	CD11c	Adhesion
^{155}Gd	Gadolinium	WM59	BD	CD31	Adhesion
^{175}Lu	Lutetium	A1	Biolegend	CD39	Immune regulation
^{173}Yb	Ytterbium	AD2	Biolegend	CD73	Immune regulation
^{162}Dy	Dysprosium	L307.4	BD	CD80	Immune regulation
^{156}Gd	Gadolinium	IT2.2	BD	CD86	Immune regulation
139La	Lanthanum	15-2	Biolegend	CD206	Immune Regulation
^{152}Sm	Samarium	5-271	Biolegend	CD36	Phagocytosis & Clearance
^{153}Eu	Europium	KP1	Biolegend	CD68	Phagocytosis & Clearance
